# Epileptic Seizure Provoked by Bone Metastasis of Chronic Lymphoid Leukemia and Merkel Cell Carcinoma

**DOI:** 10.1155/2020/4318638

**Published:** 2020-10-27

**Authors:** András Folyovich, Angéla Majoros, Tamás Jarecsny, Gitta Pánczél, Zsuzsanna Pápai, Gábor Rudas, Lajos Kozák, Gábor Barna, Katalin A. Béres-Molnár, Károly Vadasdi, Gabriella Liszkay, Eszter Horváth, Gergely Toldi

**Affiliations:** ^1^Department of Neurology and Stroke, Szent János Hospital, Budapest, Hungary; ^2^National Institute of Oncology, Budapest, Hungary; ^3^Military Hospital, Budapest, Hungary; ^4^MR Research Centre, Semmelweis University, Budapest, Hungary; ^5^First Department of Pathology and Experimental Cancer Research, Semmelweis University, Budapest, Hungary; ^6^Department of Laboratory Medicine, Semmelweis University, Budapest, Hungary

## Abstract

**Background:**

Merkel cell carcinoma (MCC) is a rare primary neuroendocrine cutaneous tumor, rarely metastasizing to the brain. Chronic lymphoid leukemia (CLL) is a disease predisposing to MCC. According to previous reports, headache and focal neurological deficits suggest disease progression to the brain. We present a patient with MCC whose seizure was not elicited by a cerebral metastasis, but by bone metastases compressing the brain. *Case Presentation*. A 62-year-old female patient had a history of CLL. A lesion with the appearance of an atheroma was removed from the right upper arm. Histology confirmed the diagnosis of MCC. She was admitted to the neurology department with her first GM seizure. The cranial MRI/MRA showed bone metastases in the right parietal and both frontal areas, compressing the brain. Flow cytometry of CSF did not reveal metastasis of MCC.

**Conclusions:**

The case history of the patient was unique even among the rare cases of MCC with neurological involvement. The seizure was not elicited by a cerebral metastasis, but by bone metastases compressing the brain. In addition to patient history, clinical presentation and radiological findings enabled a suspected diagnosis of skull metastasis of MCC compressing the brain, causing symptomatic epileptic seizures.

## 1. Introduction

Merkel cell carcinoma (MCC) is a rare and extremely aggressive neuroendocrine malignancy. Both clinically and histologically, it is difficult to differentiate it from neuroendocrine carcinomas, skin metastasis of small cell lung cancer, small cell malignant melanoma, malignant lymphoma, atypical keratoacanthoma, and leiomyosarcoma. In addition to patient history, clinical and radiological findings as well as immunohistochemistry contribute to a definitive diagnosis. Due to the above reasons, it is a significant challenge for the neurologist to diagnose this very rare and malignant disease in a timely manner which needs rapid treatment to provide a chance of recovery.

## 2. Case Presentation

The 62-year-old female patient's chronic lymphoid leukemia (CLL) was diagnosed 10 years ago and was nonprogressive following chemotherapy. The only other past medical history of note was laparoscopic cholecystectomy in 2006.

A lesion with the appearance of an atheroma was removed from the right upper arm 2 years ago. Histology with immunohistochemistry confirmed the diagnosis of MCC, and the patient received combined chemotherapy based on National Comprehensive Cancer Network (NCCN) guidelines. This was discontinued due to bone marrow depression. The lesion recurred 7 months later and was reoperated. At this time, the patient also had right axillary block dissection due to the local recurrence of MCC and its metastasis to the axillary lymph nodes, confirmed by aspiration cytology, showing metastases from both CLL and MCC in the lymph nodes. Postoperative radiotherapy was performed, but the patient's condition deteriorated. Progressively, a right axillary soft tissue conglomerate mass infiltrated the right lateral thoracic wall, and pathologic upper mediastinal and parasternal lymph nodes appeared. These all showed metastases from MCC. Chemotherapy following the ECO regimen (epirubicin (E) given weekly in combination with cyclophosphamide (C) and vincristin (O) applied during a 3-week interval) was reinitiated due to clinical progression, and the last chemotherapy session (vincristin) was administered 3 days before the patient was admitted to our Department of Neurology due to her first epileptic seizure (GM). The patient complained about headache during the week before admission.

### 2.1. General Medical State

BP: 115/75 mmHg and HR: 75/min; no bruit over carotid or subclavian arteries; an oblique surgical scar with primary healing on the right upper arm; liver edge at the costal margin. No other abnormality was found during physical examination.

### 2.2. Neurological State

No abnormality was found.

### 2.3. Mental State

No abnormality was found.

### 2.4. Laboratory Findings

WBC: 11.48 10^*∗*^9/L; neutrophils (%): 5.9, ratio of stab cells: 1%; lymphocytes (%): 86.4%; monocytes (%): 1.0%; eosinophiles (%): 0.1%; basophiles (%): 0.4%; RBC: 3.90 10^*∗*^12/L; Hgb: 112 g/L; Hct: 0.335 L/L; platelets: 85 10^*∗*^9/L; blood smear: Gumprecth's shadows.

Serum glucose, creatinine, and electrolyte levels were normal. Gamma-GT: 24 U/L; alkaline phosphatase: 162 U/L; LDH: 1768 U/L; amylase: 75 U/L; CRP: 1.59 mg/L.

### 2.5. Cranial CT

**Figure 1:**
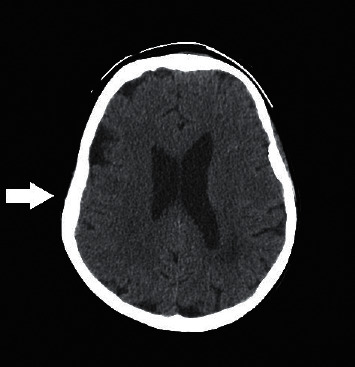
Cranial CT showing marked cerebral and cerebellar atrophy.

Cranial CT showed a 37 × 9 mm hypodensity in the right parietal region, at the posterior horn of the lateral ventricle, with marked cerebral and cerebellar atrophy (Figure 1).

### 2.6. Cranial MR

**Figure 2:**
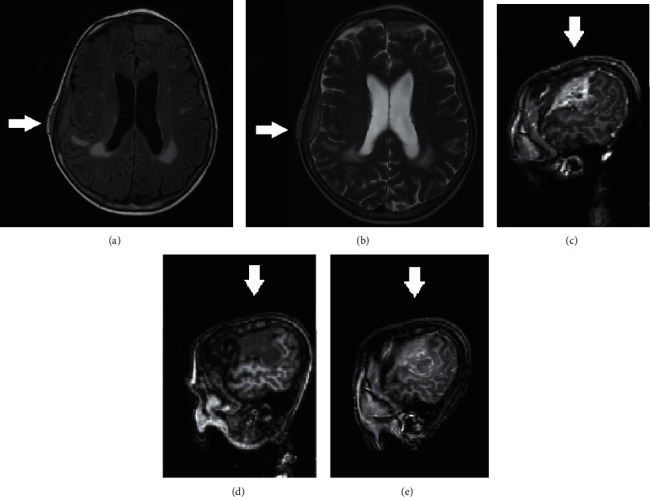
Cranial MR showing a solid epidural 2 × 2.8 × 5 cm mass, originating from the bone, in the right parietal region with (a) homogeneously low signal intensity on T1-weighted images and (b) homogeneously high signal intensity on T2 and FLAIR images. (c–e) A 6.2 × 3.2 × 1.3 cm mass, infiltrating the bone, with similar structure and contrast enhancement is seen in mainly the left frontal area.

Cranial MR showed a solid epidural of 2 × 2.8 × 5 cm mass, originating from the bone, in the right parietal region, showing homogeneously low signal intensity on T1-weighted images (Figure 2(a)) and homogeneously high signal intensity on T2 and FLAIR images (Figure 2(b)), with an intense contrast enhancement (mainly) in the peripheral zone and smaller necrotic areas in the central zone. Moderate oedema around the mass is present, with higher apparent diffusion coefficient (ADC) value on the ADC images. A 6.2 × 3.2 × 1.3 cm mass, infiltrating the bone, with similar structure and contrast enhancement is seen in mainly the left frontal area (Figures 2(c)–2(e)). The mass shows higher ADC values in diffusion images. Lacunar encephalopathy and cerebral and cerebellar atrophy were also noted.

### 2.7. EEG

EEG showed partially rhythmic activity of 7–9 Hz and 20–40 *μ*V with irregular waves of 3–4 Hz arising on the right side. Impression: organic dysfunction of the right hemisphere.

### 2.8. Lumbar Cerebrospinal Fluid (CSF)

Clear and transparent; cell count: 5 WBC/microliter; total protein: 368 mg/L; CSF glucose: 3.8 mmol/L; serum glucose (simultaneous): 7.5 mmol/L.

CSF cytology: few monocytoid cells and leukocytes. No tumor cells are detected in the sample.

CSF immunology: intact blood-liquor barrier. Intrathecal IgG synthesis is not detected, but oligoclonal gammopathy (OGP) is found.

Flow cytometry of CSF did not reveal metastasis of MCC. CLL was only present in peripheral blood, where 50% of the cells showed a corresponding flow cytometry phenotype.

Based on the clinical picture, the patient was started on carbamazepine. She did not have further epileptic seizures and did not develop any neurological deficit. Metastasis of MCC to the skull (bilateral frontal bones and right parietal bone) was diagnosed based on clinical and radiological presentation and patient history. No histology was performed from the skull mass.

The patient died 3 months later. The cause of death was multiple metastases of MCC.

## 3. Discussion

MCC is a rare and extremely aggressive neuroendocrine malignancy, presenting as a solitary, painless, thick, livid, and erythematous nodule, mostly on skin areas exposed to sunlight. First, it gives metastases to regional lymph nodes and then to distant organs. MCC is often associated with malignant hematologic diseases, such as CLL [1]. Recently, a polyomavirus was identified, likely to be involved in the pathogenesis of MCC [2]. Genetic instability and impaired immune defence due to the primary malignancy contribute to the development of other solid tumors, which may explain the coexistence of MCC with CLL. In addition to conventional radiochemotherapy, novel therapeutic options include treatment with somatostatin analogues in neuroendocrine tumors expressing somatostatin receptors, monoclonal antibody therapy in tumors expressing CD56 antigens, and anti-PD-L1 antibody therapy.

The case history of our patient is unique even among rare cases of MCC giving metastases to the brain [3, 4] due to the following factors:

1. She had a history of proven CLL
2. The seizure was not elicited by a cerebral metastasis, but by bone metastases compressing the brain

In addition to patient history, clinical presentation and radiological findings enabled the suspected diagnosis of skull metastasis of MCC compressing the brain, causing symptomatic epileptic seizures. Bone metastasis from CLL was unlikely, as solitary bone lesions in the context of CLL are believed to result from either Richter's transformation or metastasis from another primary malignancy [5], in this case, from MCC. Therefore, previous oncological diagnoses and imaging are of key importance in clarifying the origins of solitary bone lesions of the skull.

## Abbreviations

ADC: Apparent diffusion coefficient
CLL: Chronic lymphoid leukemia
CSF: Cerebrospinal fluid
MCC: Merkel cell carcinoma.
